# Radiation dose reduction with deep-learning image reconstruction for coronary computed tomography angiography

**DOI:** 10.1007/s00330-021-08367-x

**Published:** 2021-11-18

**Authors:** Dominik C. Benz, Sara Ersözlü, François L. A. Mojon, Michael Messerli, Anna K. Mitulla, Domenico Ciancone, David Kenkel, Jan A. Schaab, Catherine Gebhard, Aju P. Pazhenkottil, Philipp A. Kaufmann, Ronny R. Buechel

**Affiliations:** Department of Nuclear Medicine, Cardiac Imaging, University and University Hospital Zurich, Ramistrasse 100, CH-8091 Zurich, Switzerland

**Keywords:** Coronary angiography, Prospective studies, Deep learning, Radiation dosage, Plaque, Atherosclerotic

## Abstract

**Objectives:**

Deep-learning image reconstruction (DLIR) offers unique opportunities for reducing image noise without degrading image quality or diagnostic accuracy in coronary CT angiography (CCTA). The present study aimed at exploiting the capabilities of DLIR to reduce radiation dose and assess its impact on stenosis severity, plaque composition analysis, and plaque volume quantification.

**Methods:**

This prospective study includes 50 patients who underwent two sequential CCTA scans at normal-dose (ND) and lower-dose (LD). ND scans were reconstructed with Adaptive Statistical Iterative Reconstruction-Veo (ASiR-V) 100%, and LD scans with DLIR. Image noise (in Hounsfield units, HU) and quantitative plaque volumes (in mm^3^) were assessed quantitatively. Stenosis severity was visually categorized into no stenosis (0%), stenosis (< 20%, 20–50%, 51–70%, 71–90%, 91–99%), and occlusion (100%). Plaque composition was classified as calcified, non-calcified, or mixed.

**Results:**

Reduction of radiation dose from ND scans with ASiR-V 100% to LD scans with DLIR at the highest level (DLIR-H; 1.4 mSv vs. 0.8 mSv, *p* < 0.001) had no impact on image noise (28 vs. 27 HU, *p* = 0.598). Reliability of stenosis severity and plaque composition was excellent between ND scans with ASiR-V 100% and LD scans with DLIR-H (intraclass correlation coefficients of 0.995 and 0.974, respectively). Comparison of plaque volumes using Bland–Altman analysis revealed a mean difference of − 0.8 mm^3^ (± 2.5 mm^3^) and limits of agreement between − 5.8 and + 4.1 mm^3^.

**Conclusion:**

DLIR enables a reduction in radiation dose from CCTA by 43% without significant impact on image noise, stenosis severity, plaque composition, and quantitative plaque volume.

**Key Points:**

•*Deep-learning image reconstruction (DLIR) enables radiation dose reduction by over 40% for coronary computed tomography angiography (CCTA)*.

•*Image noise remains unchanged between a normal-dose CCTA reconstructed by ASiR-V and a lower-dose CCTA reconstructed by DLIR*.

•*There is no impact on the assessment of stenosis severity, plaque composition, and quantitative plaque volume between the two scans*.

**Supplementary Information:**

The online version contains supplementary material available at 10.1007/s00330-021-08367-x.

## Introduction

Coronary computed tomography angiography (CCTA) is a widely established diagnostic modality to improve risk stratification and management of patients with coronary artery disease (CAD) beyond ischemia testing [1, 2]. Following its widespread clinical application, the burden of radiation exposure led to the development of various strategies to enable low-dose CCTA [3, 4]. Paralleled by refinements in computed tomography (CT) hardware technology, iterative reconstruction methods have been developed by various vendors that allow for a reduction of image noise and, consequently, enable further dose reduction [5, 6]. However, iterative reconstruction algorithms have limitations. Third- and fourth-generation algorithms have become increasingly complex, incorporating a multitude of scanner-based and patient-derived parameters, requiring more and more computational power and rendering image reconstruction time-consuming. The additional complexity conferred by the increasing detector size of newest-generation CT scanners constitutes another limitation that, in some instances, renders application of iterative reconstruction difficult or even impossible. On the other hand, a plastic-like, blotchy image appearance at high levels affects and limits the evaluation and arguably also the interpretation of CT images for all iterative reconstruction methods [7]. The use of artificial intelligence to facilitate or even bypass the demanding iterative reconstruction has recently been proposed, and the potential of deep convolution neural networks for improving CT image reconstruction has been investigated [8]. These networks are optimized in the training process by minimizing differences between their output and the ideal training sample [9]. In a clinical setting, such a deep-learning image reconstruction (DLIR) algorithm has been shown to significantly reduce noise by 43% and improve image quality by 62% at equal diagnostic accuracy [10]. The aim of the present study was to assess the dose reduction capabilities of DLIR. We hypothesized that reduction of the tube voltage (and hence radiation dose) by 40% and application of DLIR instead of conventional Adaptive Statistical Iterative Reconstruction-Veo (ASiR-V) would (a) result in comparable image noise, (b) no significant impairment of stenosis severity or plaque composition assessment, and (c) no significant influence on the quantification of plaque volumes.

## Materials and methods

### Study population

Fifty consecutive patients who were referred for the assessment of known or suspected CAD with contrast-enhanced CCTA were prospectively enrolled to undergo an additional lower-dose (LD) contrast-enhanced CCTA if none of the following exclusion criteria was present: pregnancy, breast-feeding, hypersensitivity to iodinated contrast agent, renal failure (i.e., a glomerular filtration rate < 30 ml/min/1.73 m^2^) or age < 18 years [11, 12]. Written informed consent was obtained from all patients, and the study protocol was approved by the local ethics committee (BASEC-Nr. 2019–00533). The data underlying this article will be shared upon reasonable request to the corresponding author complying with ethical and privacy requirements.

### CCTA acquisition and post-processing

All patients underwent two sequential single-beat contrast-enhanced CCTA scans during breath-hold at inspiration with prospective ECG triggering at 75% of the R-R interval on a 256-slice CT scanner (Revolution CT, GE Healthcare). For the normal-dose (ND) CCTA scan, tube voltage and tube current were adapted to body mass index (BMI) as previously reported [11]. Subsequently, the LD CCTA scan was performed with 60% of the tube current used for the ND CCTA scan (i.e., based on a previous clinical study demonstrating a noise reduction of 43% by DLIR compared to ASiR-V without affecting diagnostic accuracy [10]).

Up to 30 mg of metoprolol (Beloc Zok, Astra Zeneca) was administered intravenously prior to the ND CCTA scan if the heart rate was higher than 65 beats/min to obtain optimal image quality [11]. Patients received 0.4 mg of sublingual isosorbide dinitrate (Isoket, Schwarz Pharma) 2–3 min before the ND CCTA scan. Iodixanol (Visipaque 320, 320 mg/ml, GE Healthcare) was injected into an antecubital vein followed by 50 ml saline solution via an 18-gauge catheter. For both CCTA scans, contrast agent volume and flow rate were adapted to BMI as previously reported [13]. Collimation of 256 × 0.625 mm with a z-coverage of 12–16 cm was used with a display field of view of 25 cm. All scans were acquired in high-resolution mode with an in-plane spatial resolution of 0.23 × 0.23 mm. Gantry rotation time was 280 ms.

ND CCTA datasets were reconstructed using ASiR-V at a level of 70% and of 100%. The LD CCTA datasets were reconstructed using DLIR (TrueFidelity, GE Healthcare) at the highest level (DLIR-H) from the three reconstruction strength levels (low, medium, high) provided for controlling the amount of noise reduction. DLIR employs deep convolutional neural networks–based models to pattern high-dose filtered back projection (FBP) image texture with low noise and high resolution from millions of trained parameters [14]. All image reconstructions have been performed locally on the scanner console.

For each patient, an unenhanced CT for calculation of the calcium score was acquired a few minutes prior to the contrast-enhanced CT scans on the same CT scanner (Revolution CT) using the following scan parameters: prospective ECG triggering, 2.5-mm slice thickness, 120-kV tube voltage, and 200-mA tube current, as previously reported [15].

The dose-length product multiplied by a conversion factor of 0.026 mSv × mGy^−1^ × cm^−1^ determined effective radiation dose exposure from CCTA [16].

### Quantitative image analysis

Quantitative image assessment was performed by a single reader. On a dedicated workstation (Advantage Workstation 4.7, GE Healthcare), for all reconstructions and in every patient, the aortic root was examined at the level of the left main coronary artery on an axial image using a region of interest (ROI) with a 20-mm diameter to measure mean attenuation (representing signal) and its standard deviation (SD, representing noise) in Hounsfield units (HU). From these measurements, the signal-to-noise ratio (SNR) was calculated. Similarly, measurements of mean attenuation in the proximal left main artery (LMA) and right coronary artery (RCA) were obtained using a ROI with a 2-mm diameter on axial images, and due care was taken to avoid calcifications and streak artefacts. Finally, a ROI with a 2-mm diameter was placed in the adjacent perivascular tissue to measure the vessel contrast expressed as the difference in mean attenuation in HU between the contrast-enhanced vessel and the adjacent perivascular tissue. The obtained measurements were used to calculate the contrast-to-noise ratio (CNR). For this purpose, noise was defined as the standard deviation of the attenuation in the aortic root.

### Stenosis severity and plaque composition

For the CCTA datasets reconstructed with ASiR-V 70%, ASiR-V 100%, and DLIR-H, a blinded reader visually evaluated the stenosis severity (as a percentage of the luminal vessel diameter) as well as the plaque composition in all coronary arteries (left main, left anterior descending, left circumflex, and right coronary artery) and side branches (diagonal, left marginal and posterolateral branches, as well as posterior descending artery) on a dedicated workstation as per clinical routine (Advantage Workstation, GE Healthcare). Luminal diameter was categorized as no stenosis (0%), different degrees of stenosis (i.e., < 20%, 20–50%, 51–70%, 71–90%, 91–99%), or occlusion (100%). Plaque composition was categorized into calcified, non-calcified, or mixed plaque.

### Quantitative plaque volume

For the CCTA datasets reconstructed with ASiR-V 70%, ASiR-V 100%, and DLIR-H, a blinded reader manually performed multiplanar volumetry to calculate the plaque volumes (in mm^3^) using the NIH-supported open-source software 3D Slicer (version 4.10.0, www.slicer.org) in all coronary arteries and side branches. The plaque volume of each coronary plaque was obtained and then summated to calculate the total plaque volume per patient.

### Statistical analysis

Quantitative data were expressed as mean ± standard deviation (SD) or median with the 25^th^ and 75^th^ percentile, where appropriate. The data were tested for normal distribution using the Kolmogorov–Smirnov test. Parameters derived from the different image reconstructions were compared using repeated measures of analysis of variance (ANOVA). Post hoc pairwise comparisons were adjusted for multiple comparisons by the Bonferroni correction. Intraclass correlation (ICC) analysis was applied to assess reliability of stenosis severity and plaque composition between different reconstructions. Quantitative plaque volumes obtained from the different reconstructions were compared using Bland–Altman analysis. Assuming an expected mean of differences of 17 mm^3^ and an expected standard deviation of differences of 98 mm^3^ [17] and a maximum allowed difference between methods of 307 mm^3^ [18], sample size calculations resulted in a sample size of 46 patients (type I error = 0.05, type II error = 0.05). A *p* value < 0.05 was considered statistically significant. IBM SPSS Statistics version 25 (IBM) was used for all statistical analyses.

## Results

### Baseline characteristics and scan parameters

**Fig. 1:**
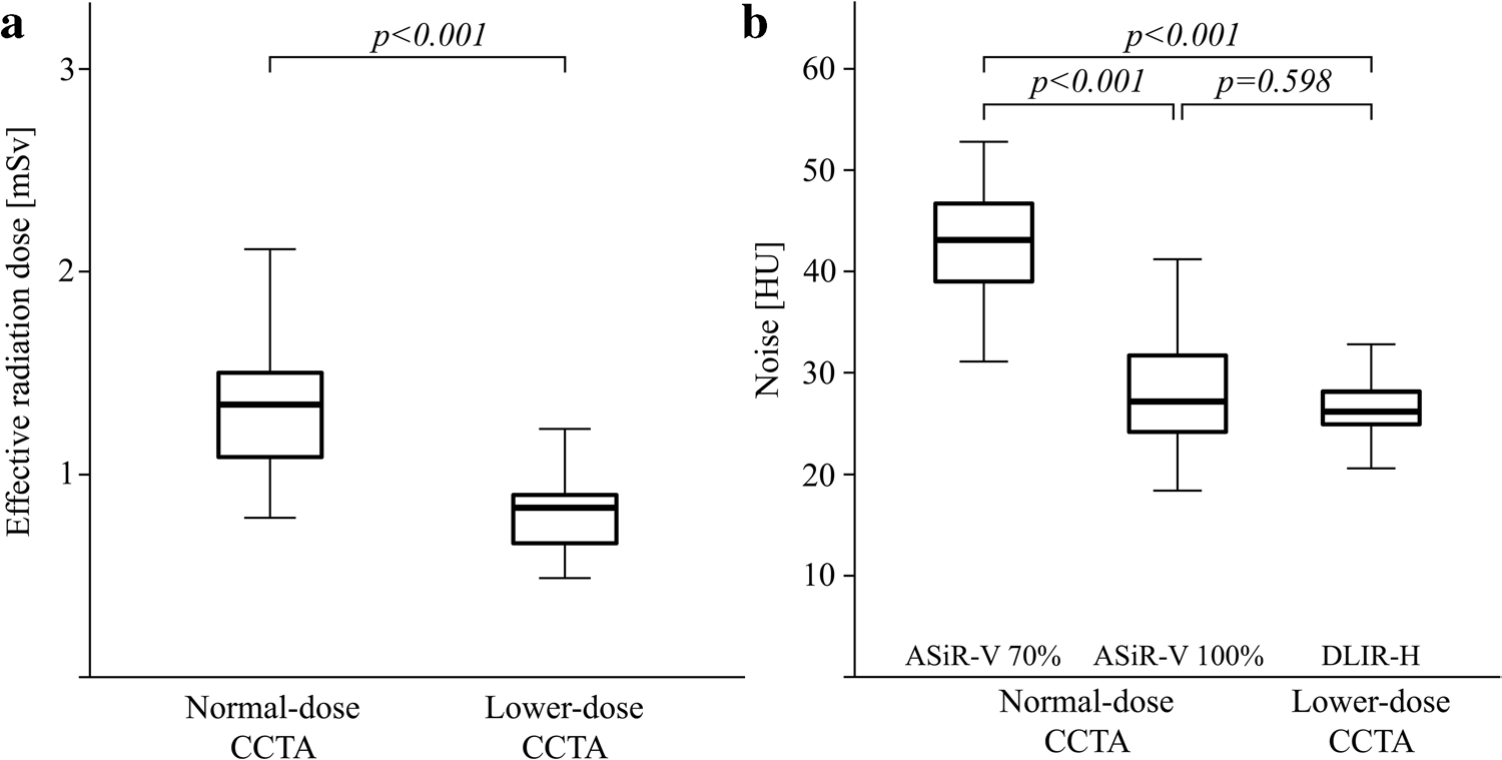
Quantitative image analysis. Box plot in panel **a** compares effective radiation dose exposure for normal-dose CCTA (median of 1.4 mSv) to lower-dose CCTA (median of 0.8 mSv). Box plot in panel **b** depicts noise in normal-dose CCTA reconstructed with ASiR-V 70% (mean of 42 HU) and with ASiR-V 100% (mean of 28 HU) as well as in lower-dose CCTA reconstructed with DLIR-H (mean of 27 HU)

A total of 50 consecutive patients were prospectively enrolled and underwent an ND and a subsequent LD CCTA scan. The baseline characteristics of the study population and the scan parameters are given in Tables 1 and 2, respectively. Of note, effective radiation dose exposure decreased significantly by 0.6 mSv (− 43%) from ND to LD CCTA (Fig. 1a).

**Table 1:** Baseline characteristics and scan parameters (*n* = 50)

Characteristic	Finding
Age (years)	59 ± 1
BMI (kg/m^2^)	27 ± 1
Cardiac risk factors	
Smoking	11 (22)
Diabetes mellitus	6 (12)
Hypertension	22 (44)
Dyslipidemia	23 (46)
Family history of CAD	21 (42)
Symptoms	
Asymptomatic	23 (46)
Typical chest pain	3 (6)
Atypical chest pain	18 (36)
Dyspnea	6 (12)
Medications	
Antithrombotic	7 (14)
Beta blocker	9 (18)
ACEi/ARB	17 (34)
Statin	16 (32)
Cardiac history	
Known CAD	1 (2)
Previous MI	0 (0)
Previous PCI	1 (2)
Previous CABG	0 (0)
Coronary artery calcium score	41 [2–174]
No coronary artery calcifications	9 (18)

Data given are absolute numbers (percentage), mean ± standard deviation or median [interquartile range]. Abbreviations: *ACEi* angiotensin-converting enzyme inhibitor, *ARB* angiotensin receptor blocker, *BMI* body mass index, *CABG* coronary artery bypass grafting, *CAD* coronary artery disease, *MI* myocardial infarction, *PCI* percutaneous coronary intervention

**Table 2:** Scan parameters

Characteristic	ND CCTA	LD CCTA	*p* value
Heart rate (beats/min)	56 [52–59]	54 [50–60]	0.09
Heart rate variability (beats/min)	7 [4–11]	8 [4–12]	0.79
Tube voltage (kVp)	100 [100–100]	100 [100–100]	NA
Tube current (mA)	439 [349–460]	264 [209–274]	< 0.001
Dose-length product (mGy*cm)	52 [42–58]	31 [25–34]	< 0.001
Effective radiation dose (mSv)	1.4 [1.1–1.5]	0.8 [0.7–0.9]	< 0.001

Data given are median [interquartile range]. Abbreviations: *CCTA* coronary computed tomography angiography, *kVp* kilovoltage peak, *NA* not available, *ND* normal-dose, *mA* milliampere, *mGy* milligray, *mSv* millisievert, *LD* low-dose

### Quantitative image analysis

An overview of the quantitative image assessment is given in Table 3. While signal intensity remained unchanged across different CCTA scans and reconstructions, all other parameters, including noise, SNR, CNR, and image quality, differed significantly. Moreover, noise was comparable between ND CCTA reconstructed with ASiR-V 100% (28 ± 6) and LD CCTA reconstructed with DLIR-H (27 ± 4) while it was significantly higher in ASiR-V 70% (42 ± 6) (Fig. 1b). Similar results were obtained for SNR and CNR.

**Table 3:** Quantitative image analysis

Variable	Normal-dose CCTA		Lower-dose CCTA	*p* value
	ASiR-V 70%	ASiR-V 100%	DLIR-H	
Signal AR (HU)	443 ± 85	443 ± 85	462 ± 76	0.198
Noise AR (HU)	42 ± 6*	28 ± 6	27 ± 4	< 0.001
SNR AR	11 ± 2*	16 ± 2	17 ± 3	< 0.001
CNR LMA	12 ± 2*	17 ± 3	19 ± 3	< 0.001
CNR RCA	11 ± 2*	17 ± 3	19 ± 3	< 0.001

Data given are mean ± standard deviation. Abbreviations: *AR* aortic root, *ASiR-V* Adaptive Statistical Iterative Reconstruction-Veo, *CNR* contrast-to-noise ratio, *DLIR-H* deep-learning image reconstruction at high level, *HU* Hounsfield units, *LMA* left main artery, *RCA* right coronary artery, *SNR* signal-to-noise ratio

Post hoc pairwise comparison with Bonferroni-adjustment for multiple testing revealed significant mean differences from DLIR-H (*) (*p* < 0.05)

### Stenosis severity and plaque composition

Seven out of 766 coronary artery segments (0.9%) were excluded from the analysis due to stents (*n* = 2) or image artefacts (*n* = 5).

Reliability of stenosis severity was excellent for all comparisons (Table 4; see supplemental material, Fig. S1A), with ICC coefficients of 0.935, 0.995, and 0.995 for ASiR-V 100% vs. ASiR-V 70%, DLIR-H vs. ASiR-V 100%, or DLIR-H vs. ASiR-V 70%, respectively.

**Table 4:** Stenosis severity, plaque composition, and quantitative plaque volumes

	ASiR-V 100% vs ASiR-V 70%	DLIR-H vs ASiR-V 100%	DLIR-H vs ASiR-V 70%
Stenosis severity			
ICC (95% CI)	0.935 (0.924, 0.943)	0.995 (0.994, 0.995)	0.995 (0.994, 0.996)
Plaque composition			
ICC (95% CI)	0.988 (0.986, 0.990)	0.974 (0.971, 0.978)	0.987 (0.985, 0.989)
Quantitative plaque volumes			
Mean bias ± SD	− 0.5 mm^3^ ± 2.4	− 0.8 mm^3^ ± 2.5	− 0.3 mm^3^ ± 2.6
LOA	− 5.2 mm^3^ and 4.1 mm^3^	− 5.8 mm^3^ and 4.1 mm^3^	− 5.5 mm^3^ and 4.8 mm^3^

Abbreviations: *ASiR-V* Adaptive Statistical Iterative Reconstruction-Veo, *DLIR-H* deep-learning image reconstruction at high level, *ICC* intraclass correlation, *LOA* limits of agreement, *SD* standard deviation

In analogy, reliability of plaque composition was also found to be excellent for all comparisons (Table 4; see supplemental material, Fig. S1B), with ICC coefficients of 0.988, 0.974, and 0.987 for ASiR-V 100% vs. ASiR-V 70%, DLIR-H vs. ASiR-V 100%, or DLIR-H vs. ASiR-V 70%, respectively.

### Quantitative plaque volumes

Agreement on quantitative plaque volume (Table 4; see supplemental material, Fig. S1C) was excellent between ASiR-V 100% and ASiR-V 70% with a mean difference of − 0.5 mm^3^ (± 2.4 mm^3^) and limits of agreement (LOA) between − 5.2 and + 4.1 mm^3^. Similarly, the agreement between DLIR-H and ASiR-V 100% was excellent with a mean difference of − 0.8 mm^3^ (± 2.5 mm^3^) and LOA between − 5.8 and + 4.1 mm^3^. Comparison of DLIR-H and ASiR-V 70% also showed excellent agreement with a mean difference of − 0.3 mm^3^ (± 2.6 mm^3^) and LOA between − 5.5 and + 4.8 mm^3^.

## Discussion

**Fig. 2:**
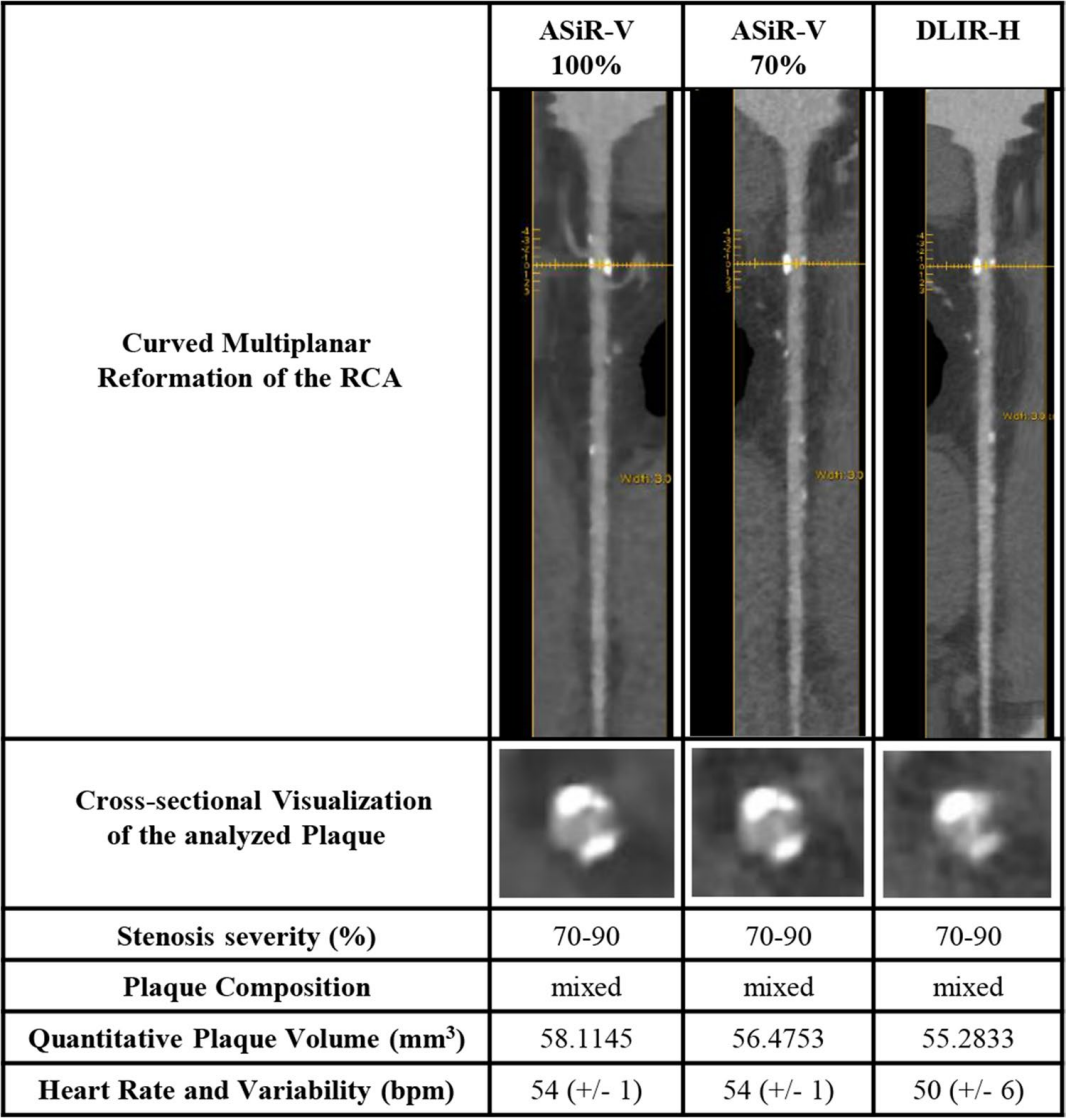
Case example. Curved multiplanar reformation of the RCA with cross-sectional visualization of the analyzed plaque is shown. ASiR-V, Adaptive Statistical Iterative Reconstruction-Veo; bpm, beats per minute; DLIR-H, deep-learning image reconstruction at high level; RCA, right coronary artery

**Fig. 3:**
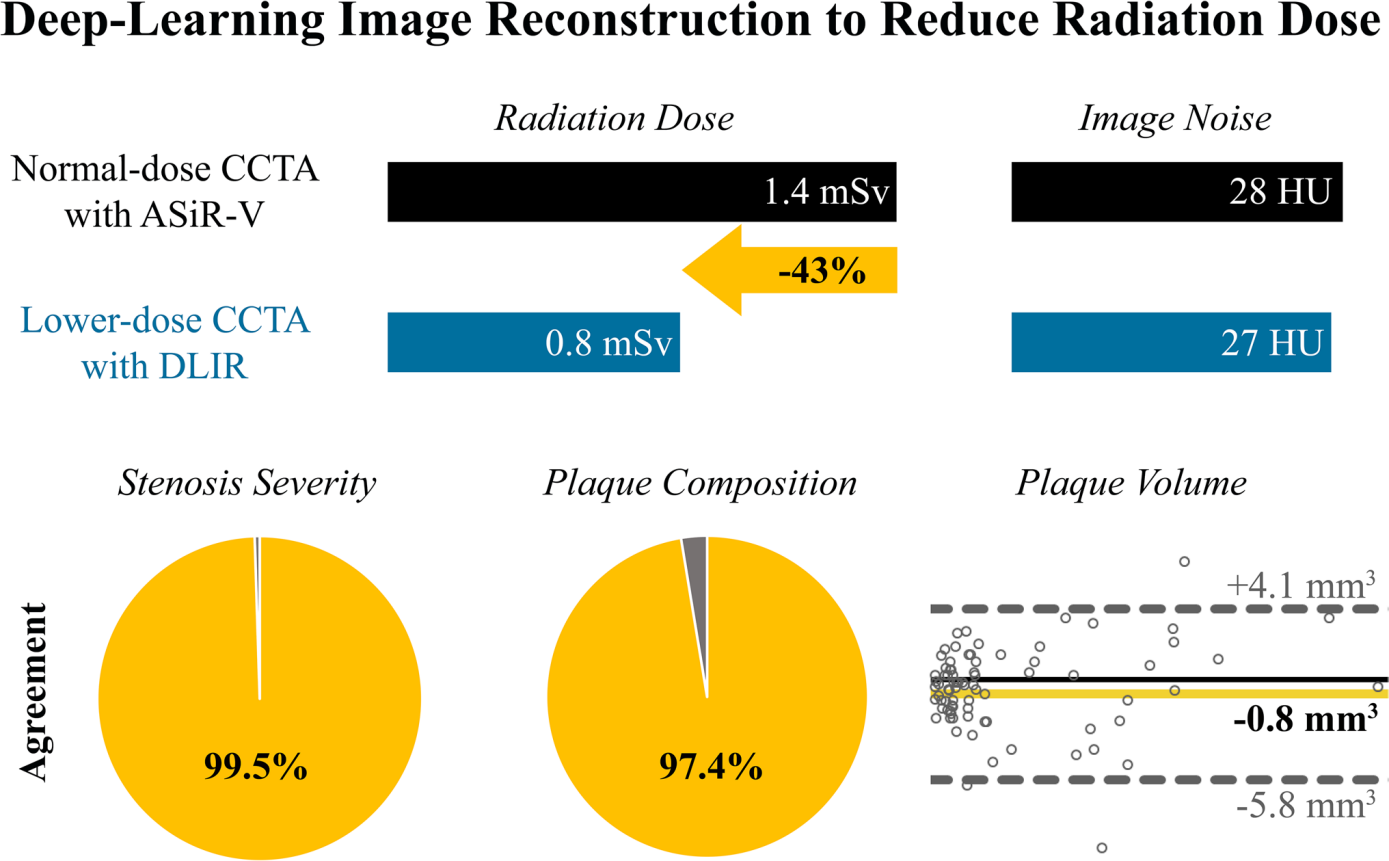
Graphical abstract. The figure summarizes the key findings of the study. Normal-dose coronary computed tomography angiography (CCTA) had a median effective radiation dose of 1.4 mSv and was reconstructed by ASiR-V. In the subsequent CCTA, radiation dose was lowered by 43% to a median of 0.8 mSv. By reconstructing the scan with DLIR, image noise remained unchanged. Similarly, agreement on stenosis severity, plaque composition, and quantitative plaque volume was excellent between the two CCTA scans. ASiR-V, Adaptive Statistical Iterative Reconstruction-Veo; DLIR, deep-learning image reconstruction

The present study demonstrates that reducing the radiation dose by 43% combined with CCTA reconstruction by DLIR instead of conventional ASiR-V yields comparable image noise and neither impacts the assessment of stenosis severity and plaque composition nor the quantification of plaque volume (Fig. 2). Of note, the statistical agreement was excellent for all comparisons (Fig. 3).

### Comparison to the literature

The present study demonstrates the feasibility of lowering radiation dose by applying DLIR in CCTA. Reduction of the radiation dose using DLIR has previously been tested in comparative studies for abdominal CT and CT urography that have both shown that a radiation dose reduction of 55–71% results in comparable subjective and objective image quality [19, 20]. For CCTA, we have already demonstrated that DLIR has favorable noise texture and superior image quality compared to conventional ASiR-V [10]. In addition, we have demonstrated the potential for radiation dose reduction in the latter study, where DLIR-H yielded a reduction in image noise of up to 43% while maintaining diagnostic accuracy [10]. Based on these results, the present study aimed at assessing more clinically relevant endpoints such as stenosis severity, plaque composition, and quantitative plaque volume to test the utility of DLIR-H for radiation dose reduction. Compared to the most advanced model-based iterative reconstruction algorithm from the same vendor (Veo, GE Healthcare) [5], DLIR has—when numerically compared to this previous publication [5]—inferior noise reduction capabilities (27 ± 4 HU vs. 21 ± 4 HU), despite higher radiation exposure (31 mGy*cm [25–34] vs. 15 mGy*cm [14–18]). On the other hand, its high demand for computational power and the time-consuming reconstruction (approximately 20 min) is a relevant trade-off of model-based iterative reconstruction. In addition to the more appealing noise texture of DLIR, its reconstruction time is less than 50 s [14].

The present study confirms recent data from a phantom study that analyzed the impact of radiation dose reduction on spatial resolution and lesion detectability in CT scans reconstructed by ASiR-V and DLIR [21]. On the one hand, the study revealed unchanged spatial resolution for DLIR when the radiation dose was reduced. By contrast, in ASiR-V (due to the non-linear and non-stationary properties of iterative reconstruction algorithms), the spatial resolution was influenced by radiation dose. On the other hand, while lesion detectability was preserved, DLIR-H allowed for a radiation dose reduction of 46–56% compared to ASiR-V 50%. Considering that the present study compared DLIR-H to slightly higher levels of ASiR-V, the notion that lowering radiation dose by 40–50% is feasible when applying DLIR seems consistent across studies.

It is worth mentioning that the DLIR algorithm is based on a deep-learning approach where the solution path cannot easily be reproduced or comprehended. In light of what may be considered a “black-box” approach, the fact that the present study documents a good agreement between the plaque volume measurements between ASiR-V and DLIR is of significance. In fact, the limits of agreement in the present study compare well with previously published inter- and intrareader variability for plaque volume assessment [22, 23]. Hence, our results increase our confidence that the DLIR algorithm does neither add nor lose any image information relevant for coronary plaque assessment.

### Clinical implications

It is estimated that about 2% of all cancers in the USA may be attributable to the radiation from CT studies [24]. While the evidence for radiation-related cancer rates is convincing in the range of 30 to 90 mSv for CT studies (and in the range of 5 to 150 mSv for atomic-bomb survivors) [25], radiation dose exposure for CCTA was below 2 mSv in the present study, and the median reduction in radiation dose achieved by DLIR was only 0.6 mSv. However, in view of the expanding use of CT for coronary artery imaging and also for myocardial perfusion imaging or structural heart disease as well as of the large variability in radiation dose exposure in clinical practice (up to 30 mSv for helical scanning) [26], the clinical value of a reduction of more than 40% enabled simply by the application of a newest-generation image reconstruction algorithm such as DLIR becomes evident.

From a clinical perspective, the finding that—despite a substantial radiation dose reduction—reconstructions with DLIR affected neither stenosis severity, plaque composition, nor quantitative plaque volume is critical. First, the classification of stenosis severity is key for risk stratification of patients with CAD [27]. Additionally, stenosis severity guides patient management: while ischemia testing is mandatory to assess the need for revascularization of lesions between 50 and 90%, patients with non-obstructive lesions do not need further work-up for ischemic heart disease [28]. On the other hand, according to current clinical guidelines, patients with lesions above 90% may be referred for revascularization without testing for hemodynamic relevance [29]. Second, the composition of the plaque (i.e., calcified, non-calcified, or mixed) has independent and incremental prognostic value over cardiovascular risk factors, calcium score, disease extent, and disease location as well as stenosis severity [30, 31]. Specifically, the presence of mixed plaques is associated with a ten-fold increase of major adverse cardiovascular events. The reliable identification of these plaques, therefore, is of utmost importance. Third, semiautomated quantification of plaque volume incrementally improves risk stratification over the clinical risk profile and conventional CT reading [32]. Although previous studies did not identify any relevant impact of image reconstruction on quantitative plaque volume within a single scan [33, 34], its reliability is substantially affected by numerous factors in pre-processing (e.g., CT scanner, CT acquisition parameters, contrast opacification) [35, 36]. Since the present study compared sequential scans acquired at different tube currents, the favorable influence of DLIR on image reconstruction of the LD CCTA (with an excellent agreement for quantitative plaque volumes between the ND and LD CCTA), consequently, has decisive clinical implications.

### Limitations

We acknowledge the following limitations: The present study did not include a comparison to an external reference standard (i.e., invasive coronary angiography, intravascular ultrasound). However, the aim of the present study was to assess the feasibility of lowering radiation dose exposure by applying DLIR. The impact of DLIR on diagnostic accuracy using invasive coronary angiography has been demonstrated previously [10]. Nonetheless, future studies should investigate the impact of DLIR on quantitative plaque volume using intravascular ultrasound or optical coherence tomography as a standard of reference. The agreement between and among readers has not been tested again as previous studies already documented excellent inter- and intrareader reliability [10, 23, 37].

## Conclusion

DLIR enables a reduction in radiation dose from CCTA by 43% without significant impact on image noise, stenosis severity, plaque composition, and quantitative plaque volume.

## Supplementary Information

Below is the link to the electronic supplementary material.

Supplementary file1 (DOCX 82 KB)

## Abbreviations

ASiR-V: Adaptive Statistical Iterative Reconstruction-Veo
CNR: Contrast-to-noise ratio
DLIR: Deep-learning image reconstruction
DLIR-H: DLIR at high level
FBP: Filtered back projection
ICC: Intraclass correlation
LD: Lower-dose
LOA: Limits of agreement
ND: Normal-dose
SNR: Signal-to-noise ratio

## Declarations

### Guarantor

The scientific guarantor of this publication is Ronny R. Buechel, MD.

### Statistics and biometry

No complex statistical methods were necessary for this paper.

### Methodology

•prospective

•non-randomized

•performed at one institution
