# Supplementary material for: Radiation dose reduction with deep-learning image reconstruction for coronary computed tomography angiography
Source: Eur Radiol. 2021 Nov 18;32(4):2620–8. doi: 10.1007/s00330-021-08367-x (PMC8921160; doi:10.1007/s00330-021-08367-x)
Supplement: Supplementary file 1 — Supplementary file1 (DOCX 82 KB) [file 330_2021_8367_MOESM1_ESM.docx]

***SUPPLEMENTAL MATERIAL***


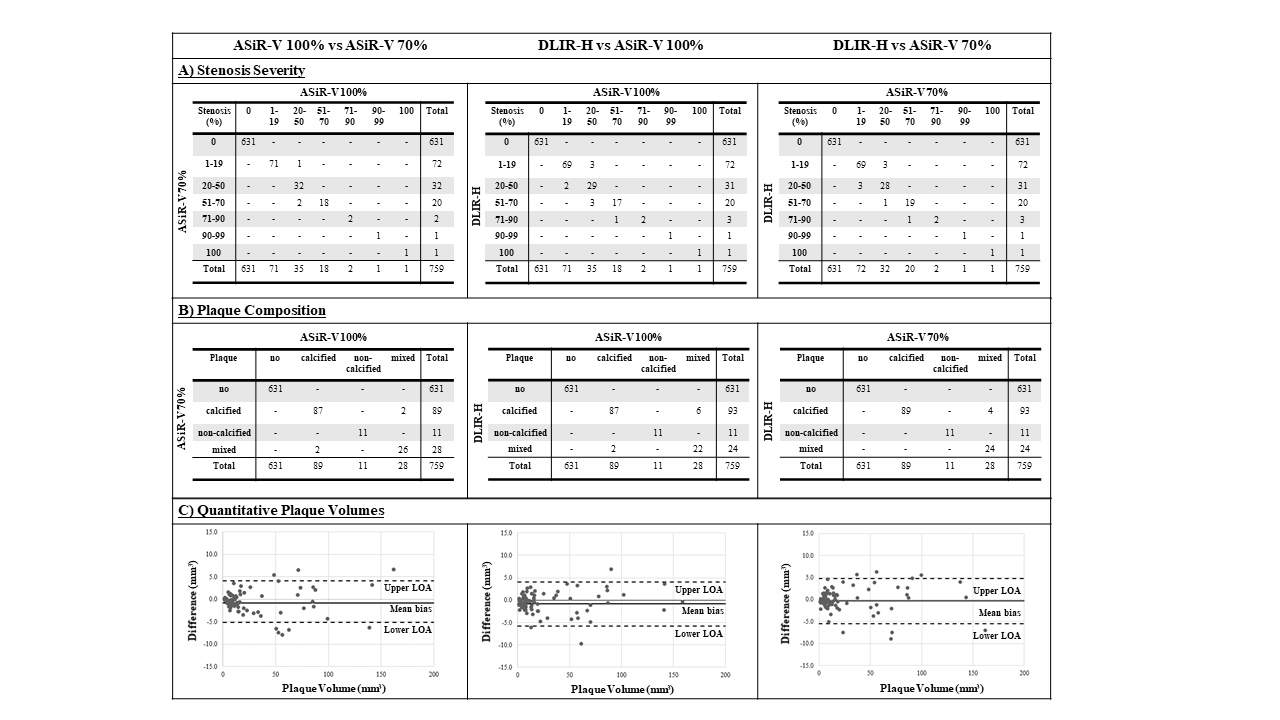


**Figure S1** **Stenosis Severity, Plaque Composition and Quantitative Plaque Volumes**

Comparisons of stenosis severity (A), plaque composition (B) and quantitative plaque volumes (C) are shown for ASiR-V 100% v.s ASiR-V 70% (left), DLIR-H vs. ASiR-V 100% (middle) and DLIR-H vs ASiR-V 70% (right). In the Bland-Altman plots, the mean bias is shown as solid lines and the upper and lower limits of agreement are represented by dashed lines.

*Abbreviations: ASiR-V = Adaptive Statistical Iterative Reconstruction-Veo, DLIR-H = Deep-Learning Image Reconstruction at high level, ICC = Intraclass correlation, LOA = limits of agreement, SD = standard deviation.*
